# Berberine regulates the Notch1/PTEN/PI3K/AKT/mTOR pathway and acts synergistically with 17-AAG and SAHA in SW480 colon cancer cells

**DOI:** 10.1080/13880209.2020.1865407

**Published:** 2021-01-08

**Authors:** Ge Li, Chuang Zhang, Wei Liang, Yanbing Zhang, Yunheng Shen, Xinhui Tian

**Affiliations:** a Institute of Interdisciplinary Integrative Medicine Research, Shanghai University of Traditional Chinese Medicine, Shanghai, PR China; b School of Pharmacy, Zhengzhou University, Zhengzhou, PR China; c School of Pharmacy, Naval Medical University, Shanghai, PR China

**Keywords:** Connectivity map, synergistic effect, mechanism

## Abstract

**Context:**

Berberine (BBR) is used to treat diarrhoea and gastroenteritis in the clinic. It was found to have anticolon cancer effects.

**Objective:**

To study the anticolon cancer mechanism of BBR by connectivity map (CMAP) analysis.

**Materials and methods:**

CMAP based mechanistic prediction was conducted by comparing gene expression profiles of 10 μM BBR treated MCF-7 cells with that of clinical drugs such as helveticoside, ianatoside C, pyrvinium, gossypol and trifluoperazine. The treatment time was 12 h and two biological replications were performed. The DMSO-treated cells were selected as a control. The interaction between 100 μM BBR and target protein was measured by cellular thermal shift assay. The protein expression of 1–9 μM BBR treated SW480 cells were measured by WB assay. Apoptosis, cell cycle arrest, mitochondrial membrane potential (MMP) of 1–9 μM BBR treated SW480 cells were measured by flow cytometry and Hoechst 33342 staining methods.

**Results:**

CMAP analysis found 14 Hsp90, HDAC, PI3K or mTOR protein inhibitors have similar functions with BBR. The experiments showed that BBR inhibited SW480 cells proliferation with IC_50_ of 3.436 μM, induced apoptosis, autophage, MMP depolarization and arrested G1 phase of cell cycle at 1.0 μM. BBR dose-dependently up-regulated PTEN, while inhibited Notch1, PI3K, Akt and mTOR proteins at 1.0–9.0 μM (*p* < 0.05). BBR also acted synergistically with Hsp90 and HDAC inhibitor (0.01 μM) in SW480 cells at 0.5 and 1.0 μM.

**Discussion and conclusions:**

The integrative gene expression-based chemical genomic method using CMAP analysis may be applicable for mechanistic studies of other multi-targets drugs.

## Introduction

Natural products and their derivatives account for 37.3% of approved antitumor drugs due to chemical structure diversity, better biocompatibility and drug-like properties (Newman and Cragg 2016). Berberine (BBR) is a natural product first isolated from the bark of *Zanthoxylon clava* herculis Linne (Rutaceae) in 1826, it is used clinically for diarrhoea and gastroenteritis. BBR was reported to have a variety of other pharmacological effects such as antitumor (Farooqi et al. 2019; Gao et al. 2019; Mohammadinejad et al. 2019; Yao et al. 2019), antidiabetic and hipolipidemic (Lan et al. 2015; Sahebkar and Watts 2017; Ma et al. 2018), diminishing liver fibrosis (Feng et al. 2009), neuroprotective (Pirmoradi et al. 2019), anti-inflammatory (Lu et al. 2019) and antioxidant (Kazaz et al. 2020). Colon cancer, the third most prevalent cancer worldwide, is increasing in individuals less than 50 years old (Siegel et al. 2019). The common treatments of colon cancer include surgery, radiotherapy, chemotherapy and molecular targeted therapy. Despite the great improvements in diagnostic and therapeutic methods, the prognosis of colon cancer remains poor. Therefore, investigation of novel drugs as well as the underlying molecular mechanisms is very necessary. Recently, intensive studies have found that BBR exerts anticolon cancer effect by interacting with AMPK (Park et al. 2012; Li et al. 2015), TGF-β (Huang, Tao et al. 2019, Huang, Wang et al. 2019), EGFR (Wang et al. 2013), ROS (Hsu et al. 2007; Wang et al. 2012), Wnt/β-catenin (Wu et al. 2012; Ruan et al. 2017), microRNAs (Huang et al. 2017; Lü et al. 2018), inflammation (Fukuda et al. 1999; Chidambara Murthy et al. 2012; Xu et al. 2012), arylamine *N*-acetyltransferase (Lin et al. 1999), glucose metabolism (Mao et al. 2018), Cl^−^ secretion (Alzamora et al. 2011) and intestinal mucosal permeability (Wu et al. 2018). The above studies indicated the significant application potential of BBR in colon cancer treatment. However, does BBR act on other unknown targets or pathways? Can it be used in combination with other clinical anticancer drugs? We innovatively apply CMAP analysis to study the anticolon cancer mechanism of BBR and obtain novel information.

The connectivity map (CMAP) is a database constructed of more than 7000 gene expression profiles of drug treated cells which can identify the connection between drugs, diseases and genes (Lamb et al. 2006). By comparing the gene expression profiles of the compound with that of 1309 FDA-approved drugs, we can quickly discover the targets of our compounds, elucidate the possible mechanisms, and find new uses of clinical drugs (Hieronymus et al. 2006; Lv et al. 2017, 2018). In this study, CMAP database (http://www.broadinstitute.org/cmap/) was applied to identify potential anticolon cancer mechanism of BBR.

## Materials and methods

### Cells, reagents and antibodies

The human colon cancer cells SW480, the human hepatoma cancer cells HuH7, QGY7701 and QGY7703, the human breast cancer cells MCF-7, the human lung cancer cells A549 and the human acute monocytic leukaemia cells THP-1 were obtained from the Cell Resource Centre, Institute of Life Sciences, Chinese Academy of Medical Science. Berberine (>98% pure) was purchased from Sigma-Aldrich (St. Louis, MO). Tanespimycin (17-AAG) and Vorinostat (SAHA) were from Medchem Express (Monmouth Junction, NJ). L-15 medium, DMEM medium and penicillin/streptomycin were from Gibco (Carlsbad, CA). Foetal bovine serum was from Biological Industries (Cromwell, CT). Hoechst 33342 dye was from Sigma-Aldrich Corp. (St. Louis, MO). Annexin V-FITC was from BD Biosciences (San Jose, CA). Cell Cycle and Apoptosis Analysis Kit was from Beyotime (Nantong, China). Protease inhibitor cocktail and BCA protein quantitation kit were from Thermo Fisher (Rockford, IL).

Antibodies specific for β-actin (#4970), Hsp70 (#4872), IKKβ (#2678), EGFR (#4267), phospho-PI3K (#4228), PI3K (#4257), phosphohospho-Akt (#4060), Akt (#9272), phosphohospho-mTOR (Ser2448) (#5536), phosphohospho-mTOR (Ser2481) (#2974), mTOR (#2983), CDK4 (#12790), cyclinD1 (#2978), LC3B (#3868), Bcl-2 (#2872), BAX (#5023), PARP (#9542), cleaved caspase-3 (#9661), cleaved caspase-9 (#9509), Jagged1 (#70109), Notch1 (#3608), NICD (#4147), Hes1 (#11988) and PTEN (#9188) were obtained from Cell Signaling Technology (Danvers, MA). Anti-Hsp90 (#ab13492) antibodies were from Abcam (Cambridge, UK). Antibodies specific for HDAC1 (sc-7872), HDAC2 (sc-7899), HDAC3 (sc-11417), HDAC4 (sc-11418), HDAC6 (sc-11420) and HDAC8 (sc-11405) were purchased from Santa Cruz Biotechnology (Dallas, TX).

### CCK-8 assay

The cytotoxicity of BBR against cancer cells was assessed by using Cell Counting Kit-8. The assay was performed in triplicate. All cells were seeded in 96-well plate at a density of 5 × 10^3^ cells/well at 37 °C. After cell attachment overnight, each well was treated with different concentrations of BBR or 0.1% DMSO for 24 h, respectively. Then, 10 μL of WST-8 solution was added to each well and the cultures were incubated for another 2 h. The absorbance was read at 450 nm by a SYNERGY microplate reader (Bio Tek, Winooski, VT) (Tian et al. 2016).

### Hoechst 33342 staining

SW480 cells were first incubated with BBR for 24 h, then the cells were stained with Hoechst 33342 for 15 min in the dark. After washed by cold PBS, the cells were observed under the fluorescence microscope at 355/465 nm. Five randomly selected areas of each group were photographed (Mondal and Bennett 2016).

### Annexin V-FITC/PI double-staining assay

The SW480 cells were seeded in six-well plates (3.5 × 10^5^ cells/well) for 24 h and then treated with BBR for another 24 h. The cells were detached with 0.05% trypsin, washed three times with cold PBS, and centrifuged at 1500 rpm for 5 min at 4 °C. Subsequently, SW480 cells were gently suspended in the binding buffer containing 5 µL (10 µg/mL) Annexin V-FITC for 15 min in the dark, then incubated with 10 µL of PI (20 µg/mL) for another 5 min. The cells were immediately analysed by flow cytometry (BD FACSCanto II) (Tian et al. 2016).

### Determination of mitochondrial membrane potential (MMP)

The SW480 cells were treated with different concentrations of BBR for 24 h, and then incubated with 5 µg/mL of rhodamine 123 for 30 min in the dark. After centrifuged at 1500 rpm for 5 min, and washed with cold PBS for three times, the cells were re-suspend in 1000 µL of PBS and analysed by using flow cytometry at 488 and 530 nm, respectively (Tian et al. 2016).

### Cell cycle analysis

SW480 cells were incubated with BBR for 24 h, and then trypsinized, washed with PBS, and fixed with 70% of ethanol at 4 °C overnight. After washed twice with PBS, the cells were incubated with 100 μL of RNase A for 30 min and then stained with 1 mL of PI for 10 min in the dark. The cell-cycle distribution was analysed by flow cytometry (Tian et al. 2016).

### Cellular thermal shift assay (CETSA)

The SW480 cells (have been incubated with BBR for 30 min) were collected, washed three times with cold PBS, and then lysed in PBS supplemented with protease inhibitors following three cycles of freeze–thaw. Cell lysates were then centrifuged at 13,000 rpm for 15 min, the supernatant was diluted with cold PBS and divided into two equal parts, with one part treated with DMSO and the other part treated with BBR (100 μM). After incubation at room temperature for 30 min, the two parts were divided into nine aliquots (60 μL) respectively, then heated at temperatures of 40, 44, 48, 52, 56, 60, 64, 68 and 72 °C for 3 min. The samples were cooled for 3 min at room temperature and kept on ice. Then, every aliquot was centrifuged and the supernate was analysed by Western blot (Lv et al. 2018).

### Western blot analysis

The SW480 cells (the interaction time of drugs and cells was 24 h) were lysed with the ice-cold RIPA buffer containing 1% protease inhibitor cocktail for 15 min and then centrifuged. Total protein concentrations of the lysates were assessed using BCA protein quantitation kit. Protein samples were separated by SDS-polyacrylamide gel electrophoresis in 6–12% gels and then transferred onto methanol activated PVDF membranes at 220 mA. The PVDF membranes were blocked with 5% skim milk for 20 min, probed with the indicated primary antibody and blotted with the secondary antibody for 1 h at room temperature. Object proteins were detected by Bio-Rad ChemiDocTM (Hercules, CA) (Lv et al. 2018).

## Results

### BBR inhibits proliferation and induces apoptosis of SW480 cells

**Figure 1.**
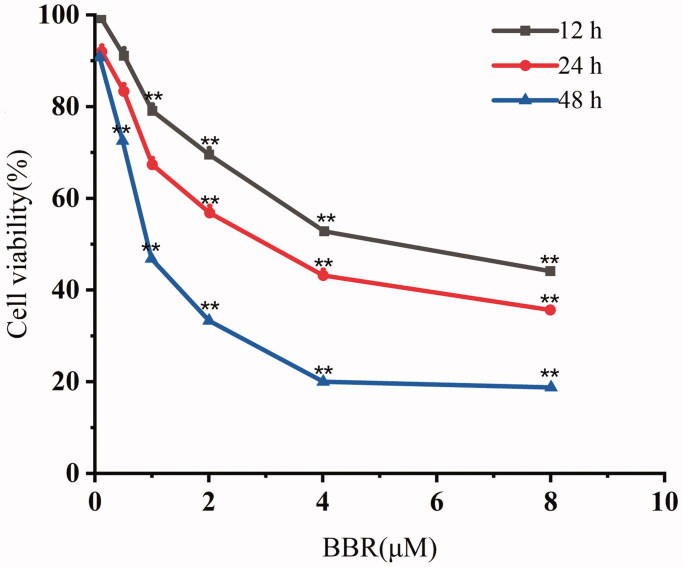
Cell viability of SW480 cells after exposure to 0–8 μM of BBR for 12, 24 and 48 h, respectively. The results are expressed as the means of three independent experiments.

We found that colon cancer cells SW480 were more sensitive to BBR treatment with IC_50_ of 3.43 μM when compared with human colon cancer cells HCT116, human breast adenocarcinoma cells MCF-7, human hepatocellular carcinoma cells QGY-7703, QGY-7701, HuH-7, human monocytic leukaemia cells THP-1 and human non-small-cell lung carcinoma cells A549 (Table 1). Further studies showed that BBR can attenuate SW480 cells proliferation in a time and dose-dependent manner at 0.5, 1, 2, 4, and 8 μM (Figure 1).

**Table 1.** Cytotoxicity effects of BBR on cancer cells (IC_50_ μM).

Cells	BBR^a^	Doxorubicin^a,b^
MCF-7	36.277 ± 1.134	0.18 ± 0.004
QGY-7701	>100	0.17 ± 0.001
QGY-7703	>100	0.53 ± 0.010
HuH7	>100	0.79 ± 0.001
THP-1	58.891 ± 2.732	0.04 ± 0.002
A549	>100	0.08 ± 0.001
HCT116	44.736 ± 1.994	0.06 ± 0.004
SW480	3.436 ± 0.193	0.14 ± 0.001

^a^ *n* = 3, means ± S.D.

^b^ Positive control.

**Figure 2.**
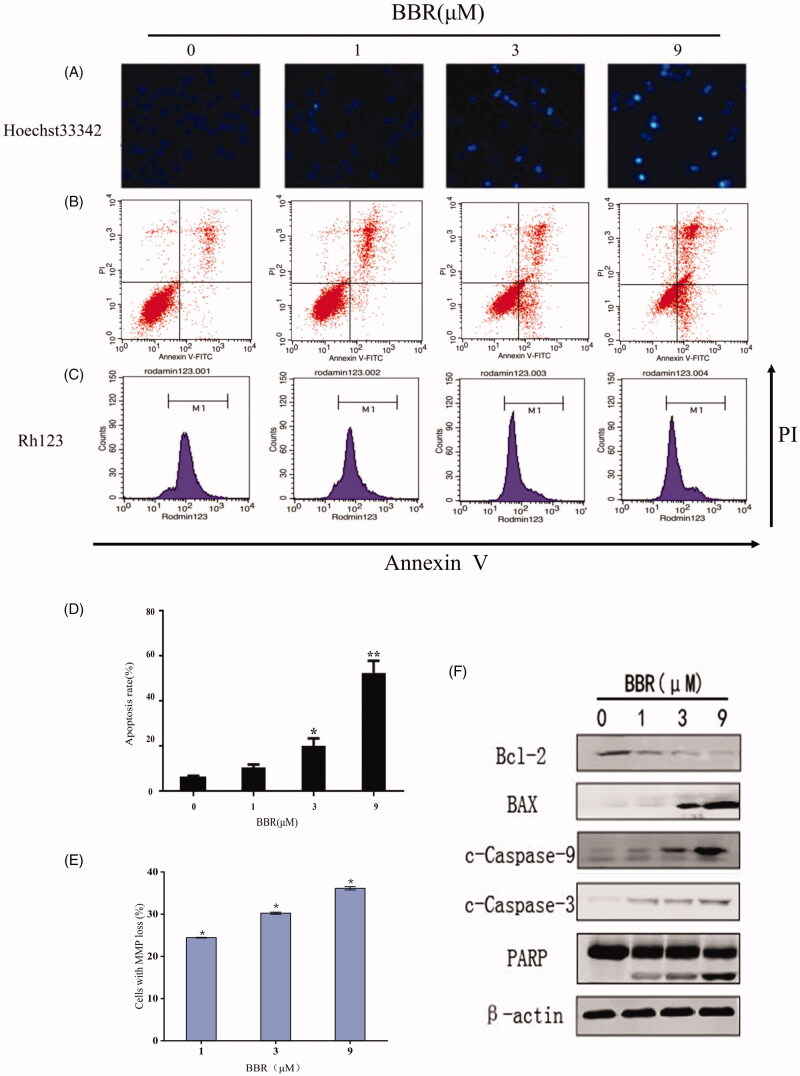
BBR induces cell apoptosis in SW480 cells after 24 h of treatment. (A) Nuclear morphological changes of SW480 cells after 33342 staining. (B) BBR induced apoptosis in SW480 cells. (C) The mitochondrial membrane potential changes of SW480 cells. (D, E) Quantification results of apoptosis and MMP changes in SW480 cells are both expressed as the means ± S.D. for three independent experiments, *p< 0.05, **p< 0.01 vs. untreated control. (F) Western blot analysis of Bcl-2, Bax, c-Caspase-9, c-Caspase-3, PARP in BBR treated SW480 cells.

Apoptosis is considered to be one of the main mechanisms to prevent the development of cancer. Thus, BBR-induced SW480 cells apoptosis was investigated by using Hoechst 33342 staining method. The blank group showed a uniformly dispersed light blue, while the BBR treated cells showed a blocky bright blue in the staining area, which is a sign of chromatin condensation. The chromatin condensation is one of the hallmarks of apoptosis. Thus, the Hoechst 33342 staining results indicated that BBR induced the apoptosis of SW480 cells at concentrations of 1–9 μM (Figure 2(A)).

Annexin V-FITC/PI double staining method was used to further study BBR-induced apoptosis in SW480 cells. The right upper and lower right quadrants of the figure represented late and early stages of cells apoptosis. As shown in Figure 2(B,D), BBR induced early and late stages of apoptosis at 1–9 μM.

**Figure 3.**
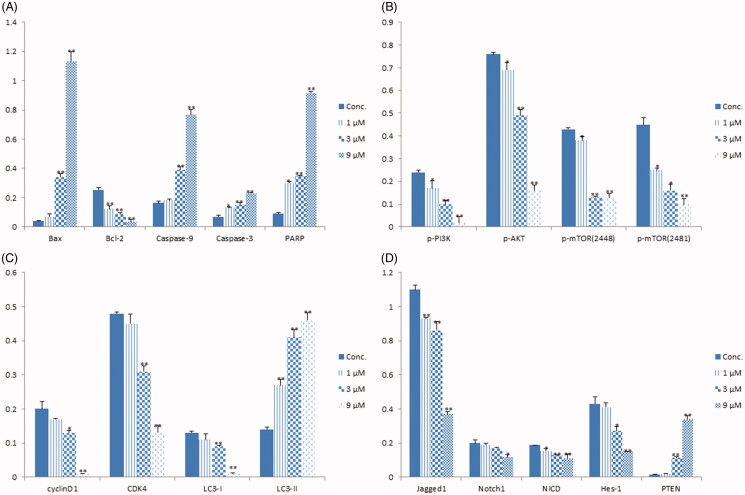
The relative densities of protein bands normalized to β-actin. (A) The relative protein densities of Bax, Bcl-2, Caspase-9, Caspase-3 and PARP. (B) The relative protein densities of p-PI3K, p-AKT, p-mTOR (2448) and p-mTOR (2481). (C) The relative protein densities of cyclinD1, CDK4, LC3-I and LC3-II. (D) The relative protein densities of Jagged1, Notch1, NICD, Hes-1 and PTEN. Data shown are mean ± standard deviation of three independent experiments. *p < 0.05, **p < 0.01 when compared to the control.

Depolarization of MMP is another characteristic of apoptosis. The decreased MMP in SW480 cells indicated that BBR-induced apoptosis was related with mitochondrial dysfunction (Figure 2(C,E)). Western blot analysis is then applied to detect whether BBR interfere with mitochondrial apoptotic pathway. First, Bcl-2 and Bax cause the mitochondrial membrane permeability changes, caspase-9 is cleaved and activated subsequently, then the apoptotic performer caspase-3 is activated, c-PARP is the cleavage substrate of caspase-3 which further regulates apoptosis. The exposure of 1–9 μM BBR to SW480 cells resulted in the up-regulating of Bax, c-caspase-9, c-caspase-3 and c-PARP, and down-regulating of Bcl-2 (Figures 2(F) and 3(A)). The above results indicated that BBR induced apoptosis follows the mitochondrial pathway.

### CMAP analysis predicts potential molecular mechanisms of BBR

Herein, CMAP was applied to identify the potential molecular mechanisms of BBR. The gene expression profiles of BBR treated cells are detected by microarray hybridization and scanning experiment using Affymetrix Human Genome U133A 2.0 chip, and a total of 526 up-regulated and 357 down-regulated genes (fold change ≥2) were obtained (Lv et al. 2017). These data were submitted into CMAP database (Version 2.0) for analysis and 14 drugs with lower *p* values and positive enrichment score were obtained (Table 2). Drugs with connectivity score closer to +1 act as higher similar to the interest, closer to −1 act as opposed to the interest, closer to 0 is unrelated to the interest. By analysing the targets of these obtained clinical drugs, we speculated that BBR may exert its antitumor activity by inhibiting Hsp90, HDAC, PI3K and mTOR proteins.

**Table 2.** Expression signatures of CMAP drugs most positively with that of BBR.

Clinical drugs	Connectivity score	*n*	*p*	Specificity	Percent non-null	Function
Helveticoside	0.517	6	0	0.0085	100	Cardiac stimulant
Ianatoside C	0.471	6	0	0.0092	100	Cardiovascular system agents
Pyrvinium	0.554	6	0	0.0093	100	Antiscolic agents
Gossypol	0.534	6	0	0.0000	100	Contraceptive agents
Trifluoperazine	0.438	16	0	0.0000	100	Dopamine antagonists
Geldanamycin	0.420	15	0	0.0054	100	Hsp90 inhibitor
Thioridazine	0.465	20	0	0.0639	100	Dopamine antagonists
Tanespimycin	0.387	62	0	0.0104	95	Hsp90 inhibitor
Prochlorperazine	0.406	16	0	0.0437	93	Dopamine antagonists
Wortmannin	0.337	18	0	0.0645	83	PI3K inhibitor
Sirolimus	0.318	44	0	0.0301	86	mTOR inhibitor
Trichostatin A	0.217	182	0	0.4123	67	HDAC inhibitor
LY-294002	0.256	61	0	0.1141	75	PI3K inhibitor
Valproic acid	0.176	57	0	0.0197	66	HDAC inhibitor

### BBR did not interact with Hsp90 and HDACs in SW480 cells

**Figure 4.**
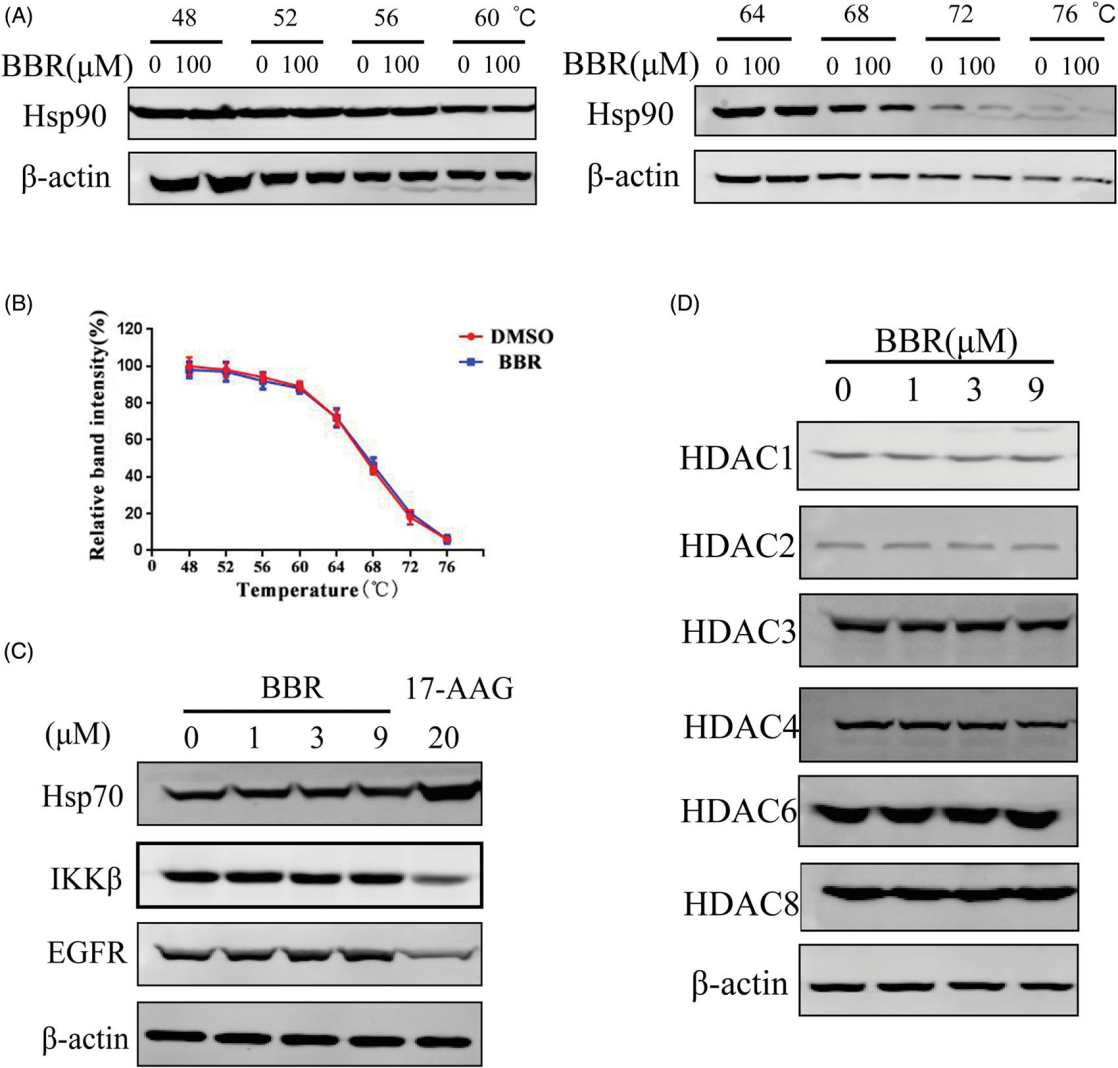
BBR did not inhibit Hsp90 and HDACs in SW480 cells. (A) The binding effect between BBR (100 μM) and Hsp90 was detected by the thermal stabilization-based CESTA method at 48–76 °C after 30 min of incubation at room temperature. (B) Hsp90 protein expression was quantified and the data are presented as means ± S.D. of three independent experiments. (C) Western blot analysis of Hsp70, IKKβ and EGFR in BBR treated SW480 cells after 24 h incubation. The Hsp90 inhibitor 17-AAG was included as positive control. (D) Western blot analysis of HDAC proteins in BBR treated SW480 cells after 24 h incubation.

The CMAP analysis revealed that BBR may be an Hsp90 inhibitor. The chaperone heat shock protein 90 (Hsp90) is responsible for the correct folding of both newly synthesized polypeptides and denatured proteins. It has hundreds of client proteins which involved in multiple oncogenic processes. Thus, Hsp90 is a promising tumour target (Neckers 2007; Schopf et al. 2017). The direct interaction between BBR and Hsp90 was examined by CETSA (Molina et al. 2013). Hsp90 protein expression decreased continuously as the incubation temperature increased. There was no significant difference between the control and BBR group, indicating that BBR did not interact directly with Hsp90 in SW480 cells (Figure 4(A,B)). Client proteins such as IKK and EGFR will be inhibited, while Hsp70 will be activated when Hsp90 is inhibited (Neckers 2007; Schopf et al. 2017). The indirect interaction between BBR and Hsp90 was then detected by analysing the above client proteins, and the results showed that BBR did not affect IKK, EGFR and Hsp70 when comparing with the positive control (Figure 4(C)). Therefore, BBR does not interact directly or indirectly with Hsp90 like a true Hsp90 inhibitor as CMAP predicted.

The CMAP analysis also revealed that BBR may function as a histone deacetylase (HDAC) inhibitor. HDAC can remove the acetyl group from histone, make the chromatin structure more compact, and silence tumour suppressor genes ultimately, thus inhibition of HDACs is considered a potential strategy for treating cancers (Xu et al. 2007). Western blot assay showed that protein levels of HDACs 1–4, 6, 8 in SW480 cells did not change as the concentrations of BBR increased, suggesting that BBR was not a HDAC inhibitor as CMAP analysis predicted (Figure 4(D)) (Mariadason 2008). Therefore, although CMAP analysis found that the gene expression profile of BBR is similar to that of Hsp90 and HDAC inhibitors, the direct or indirect interactions between them were not detected.

### BBR acted synergistically with Hsp90 and HDAC inhibitors in SW480 cells

**Figure 5.**
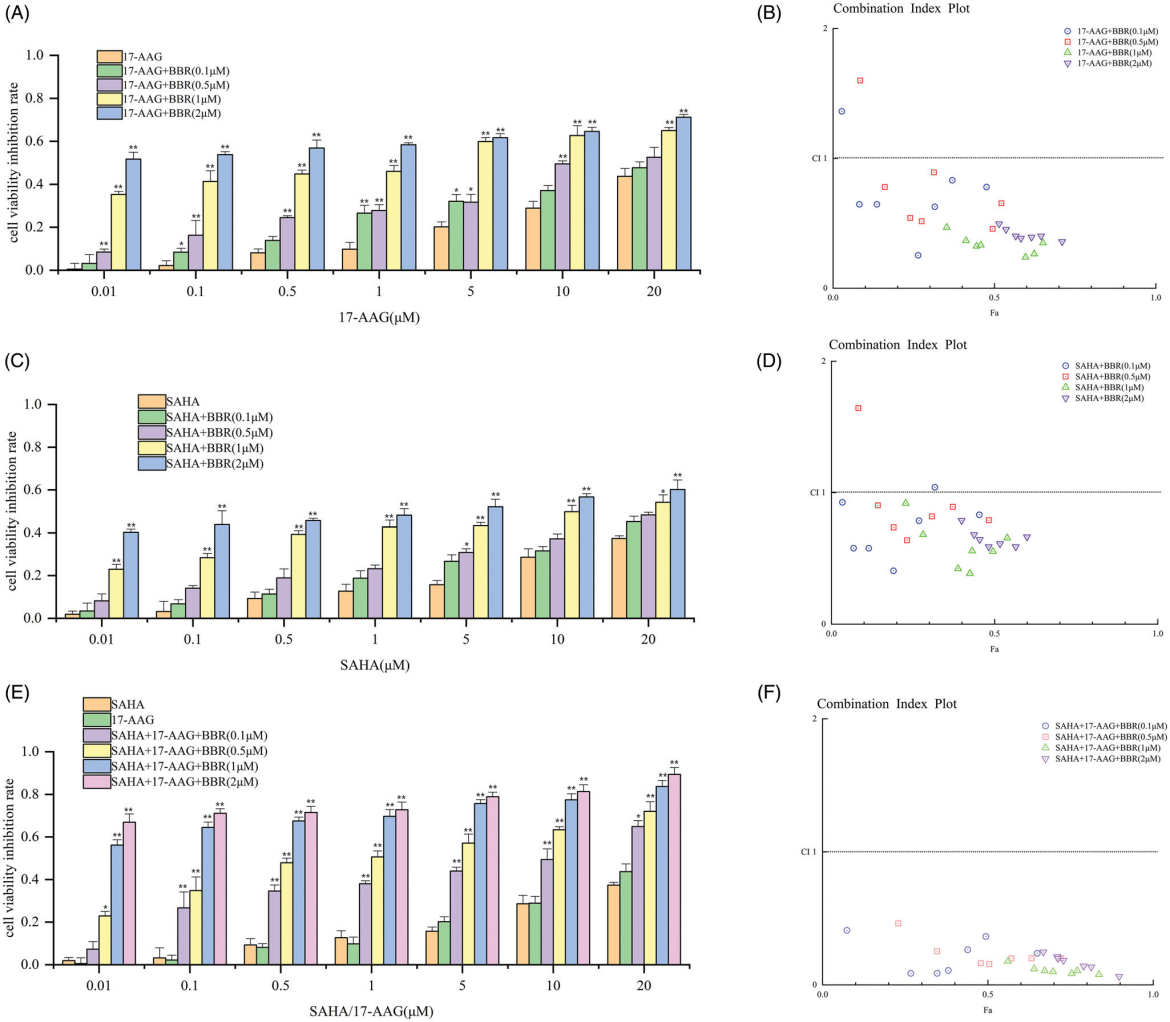
BBR enhanced the anti-proliferative activity of 17-AAG and SAHA, and the combination of the three has more potent synergistic effect on SW480 cells. The incubation time of SW480 cells and the test drugs was 24 h. (A) SW480 cells were exposed to specified concentrations of 17-AAG for 24 h in the presence of 0–2 µM of BBR. (B) The CI values of BBR and 17-AAG combinations. (C) SW480 cells were exposed to specified concentrations of SAHA for 24 h in the of 0–2 µM of BBR. (D) The CI values of BBR and SAHA combinations. (E) SW480 cells were treated with specified concentrations of 17-AAG and SAHA in the presence of 0–2 µM of BBR. (F) The CI values for BBR, 17-AAG and SAHA combinations. The above data are all presented as the means ± S.D. of three independent experiments. *p< 0.05, **p< 0.01 when comparing with 17-AAG, SAHA and both 17-AAG and SAHA treatment in the absence of BBR.

In the light of previous study has showed the synergistic effect of BBR with the clinical drug irinotecan (Yu et al. 2014), we tested whether BBR has synergistic effects with Hsp90 inhibitor 17-AAG, and HDAC inhibitor SAHA, and whether the combination of the three has synergistic effect. SW480 cells were exposed to 0–20 µM of 17-AAG, or SAHA, or both 17-AAG and SAHA for 24 h in the presence of 0–2 µM BBR. CCK-8 assay was applied to determine the cell viability. CompuSyn software was used to calculate the drug combination index (CI). Values of CI < 1, CI = 1, CI > 1 indicate synergistic, superimposed and antagonistic effect, respectively. The smaller the CI value, the more obvious the synergetic effect of the drug is. The results showed that only higher concentrations of BBR (1–2 μM) show synergistic effect with SAHA (Figure 5(A,B)). All concentrations of BBR significantly enhanced the anti-proliferative activity of 17-AAG (Figure 5(C,D)), and all of the CI values are less than 1 in Figure 5(E,F) which indicating the combination of BBR, 17-AAG and SAHA has the most potent synergistic effect.

### BBR regulates the PI3K/AKT/mTOR pathway and its upstream proteins

**Figure 6.**
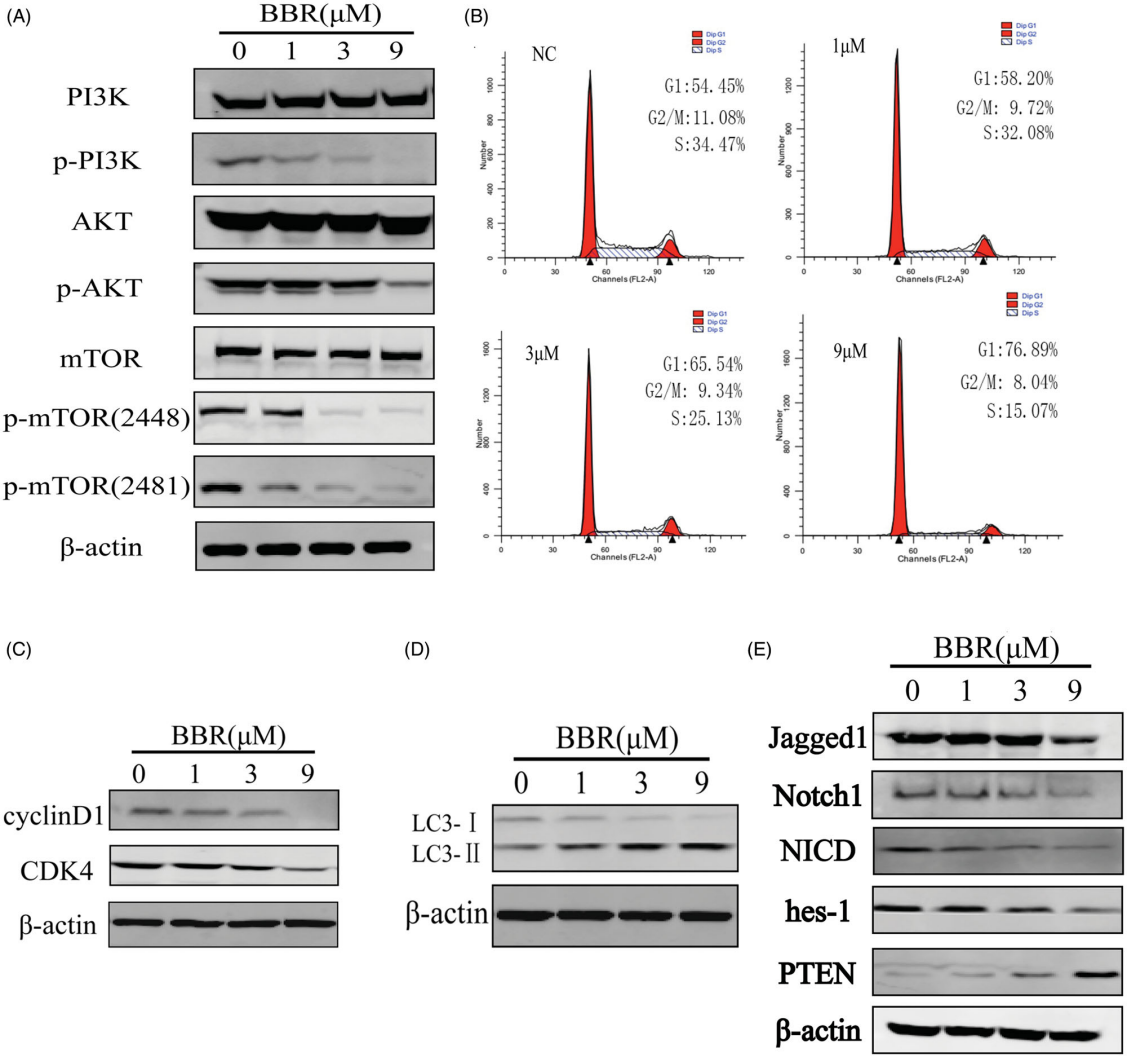
BBR targets the Notch1/PTEN/PI3K/AKT/mTOR pathway, arrests cell cycle and induces autophagy in SW480 cells after 24 h treatment. (A) The total and activated protein levels of PI3K, AKT, mTOR in BBR treated SW480 cells were determined by Western blot. (B) Flow cytometry analysis of cell cycle distribution in BBR treated SW480 cells. (C) Proteins expression of cell cycle related proteins cyclinD1 and CDK4. (D) Proteins expression of autophage related proteins LC3-I and LC3-II. (E) Proteins expression of Jagged1, Notch1, NICD, hes-1, PTEN and β-actin in BBR treated SW480 cells.

The CMAP analysis also revealed that BBR may be a PI3K or mTOR inhibitor. mTOR is the master regulator of cellular metabolism, and its deregulation is closely related with cancer. The mTOR was consisted of mTORC1 and mTORC2. PI3K/AKT signalling pathway is the well-established upstream regulator of mTORC1 (Kim and Guan 2015). The decreased levels of p-PI3K, p-Akt and p-mTOR (2448, 2481) in SW480 cells validated the CMAP prediction that BBR is the inhibitor of PI3K/AKT/m-TOR pathway (Figures 3(B) and 6(A)). Inhibition of m-TOR phosphorylation can promote autophagy. During autophagy, the cytoplasmic LC3-I enzyme dissolves a small amount of peptides into membrane type LC3-II (Tanida et al. 2008). The ratio of LC3-II/I in SW480 cells increased as the concentrations of BBR increase (Figure 3(C) and 6(D)), indicating that BBR promotes the occurrence of autophagy. In addition, it has been reported that promoting the level of autophagy could augment the arrest of cell cycle, and they are mediated by suppression of PI3K/AKT/m-TOR signalling pathway (Wang et al. 2018). DNA content analysis of SW480 cells showed that BBR induced an increase in the proportion of cells at G1 phase and a decrease at S phase, indicating a G1 phase cell cycle arrest (Figure 6(B)). The observed down-regulations of CyclinD1 and CDK4 in Figures 3(C) and 6(C)) further confirmed the G1 phase arrest of BBR. Therefore, the above studies indicated that BBR inhibits SW480 cells proliferation by inducing autophagy and cell cycle arrest under the regulation of PI3K/Akt/mTOR pathway.

Notch1 pathway proteins were reported to be highly activated in colon cancer tissues and cells, the inhibition of them may be a promising therapy for colon cancer (Zhang et al. 2010). The activation of Notch1 pathway depends on the interaction of receptors (Notch1, 2, 3, 4) and ligands (Jagged1, 2, DLL1, 3, 4) in adjacent cells. After being cleaved for three times, Notch protein released into the cytoplasm by the intracellular segment (NICD), then NICD entered the nucleus and formed transcriptional activation complex (NICD/CSL), and target proteins (Hes-1, Hes-5, Hey-1, Hey-l) are finally activated (Qiao and Wong 2009). We determined the expression of Notch1, Jagged1, NICD and Hes-1 by Western blot analysis and found that BBR inhibited them in SW480 cells (Figures 3(D) and 6(E)). Thus, BBR can significantly inhibit Notch1 pathway in SW480 cells.

Inhibition of Notch1 pathway could up-regulate the expression of PTEN. PTEN is a well-established tumour-suppressor gene which expressed in all tissues in the body, and its main target is PI3K/AKT/mTOR pathway. Through inhibiting PI3K, AKT and mTOR proteins, PTEN acts as a tumour suppressor by regulating transcription, translation, cell cycle progression, angiogenesis and stem cell self-renewal (Lee et al. 2018; Luongo et al. 2019). As observed, PTEN protein in SW480 cells was significantly increased by BBR (Figure 6(E)). Therefore, the inhibition of BBR on Notch1 pathway resulted in the increase of PTEN, following by down-regulation of PI3K/AkT/mTOR pathway, and eventually leading to cell cycle arrest and autophage in SW480 cells.

## Discussion

The natural compound BBR has been long used in Ayurvedic and Chinese medicine for a wide range of pharmacological and biochemical effects. Previous studies indicated that BBR acts on many targets and signal pathways in colon cancer cells; however, systematic understanding of genes and cellular pathways regulated by BBR is needed to define the mechanism of its anticolon cancer action. The CMAP database has been successfully used to illustrate the mechanisms of many drug candidates and discover new functions of known drugs by analysing the differentially expressed genes and comparing with the FDA approved drugs. The advantage of this chemogenomics-based technique is that it does not focus on single target gene, instead, it focuses on a series of gene expression signatures that used to query the database for similarities with known targets. Our CMAP analysis study found the positive correlations between BBR and Hsp90 inhibitor (17-AAG), HDAC inhibitor (SAHA), PI3K inhibitor and mTOR inhibitor. The interesting finding that BBR act synergistically with 17-AAG and SAHA on SW480 cells, especially the combination of the three has the most potent effect, suggested that BBR is potential to increase the expected maximal antitumor activity of 17-AAG and SAHA alone, and supported exploration of the combination therapy for colon cancer. This study also revealed a novel mechanism for the antitumor activity of BBR through the regulation of Notch1/PTEN/PI3K/AKT/mTOR pathway that regulates autophagy and cell cycle arrest. In sum, the integrative gene expression profile-based chemical genomics approach in our study provided a useful method to resolve the multi-target mechanisms of BBR. However, considering the use of gene expression profiles from BBR-treated MCF-7 cells which are commonly used in the worldwide laboratories, the variations derived from different cell types should not be neglected. There are also differences between different colon cancer cell lines and experimental conditions. Therefore, more colon cancer cell lines and corresponding non-tumour partners should be employed for mechanistic investigation. The siRNA, protein inhibition, gain-of-function and loss-of-function techniques, and *in vivo* experiments are still needed to clarify the CMAP predictions for BBR. As an improvement and supplement to our CMAP analysis, network pharmacology might help to gain more insights of anticolon cancer mechanism for BBR since it has been successfully used to evaluate many compounds with multiple targets through the integration of gene expression profiles, metabolomics and protein–protein interactions (Zhao et al. 2019).

## Conclusions

Our research provided a new method for studying the anticolon cancer mechanisms of BBR. As CMAP analysis predicted BBR promotes SW480 cells apoptosis, autophage and cell cycle arrest by inhibiting the PI3K/AKT/mTOR pathway. Further study revealed that BBR can also inhibit and promote the expression of the upstream proteins HDACs and PTEN, respectively. We first found that BBR acts synergistically with Hsp90 and HDAC inhibitors although it does not interact directly with them as CMAP analysis predicted. Our results provide a global view of the mechanisms of action of BBR.
